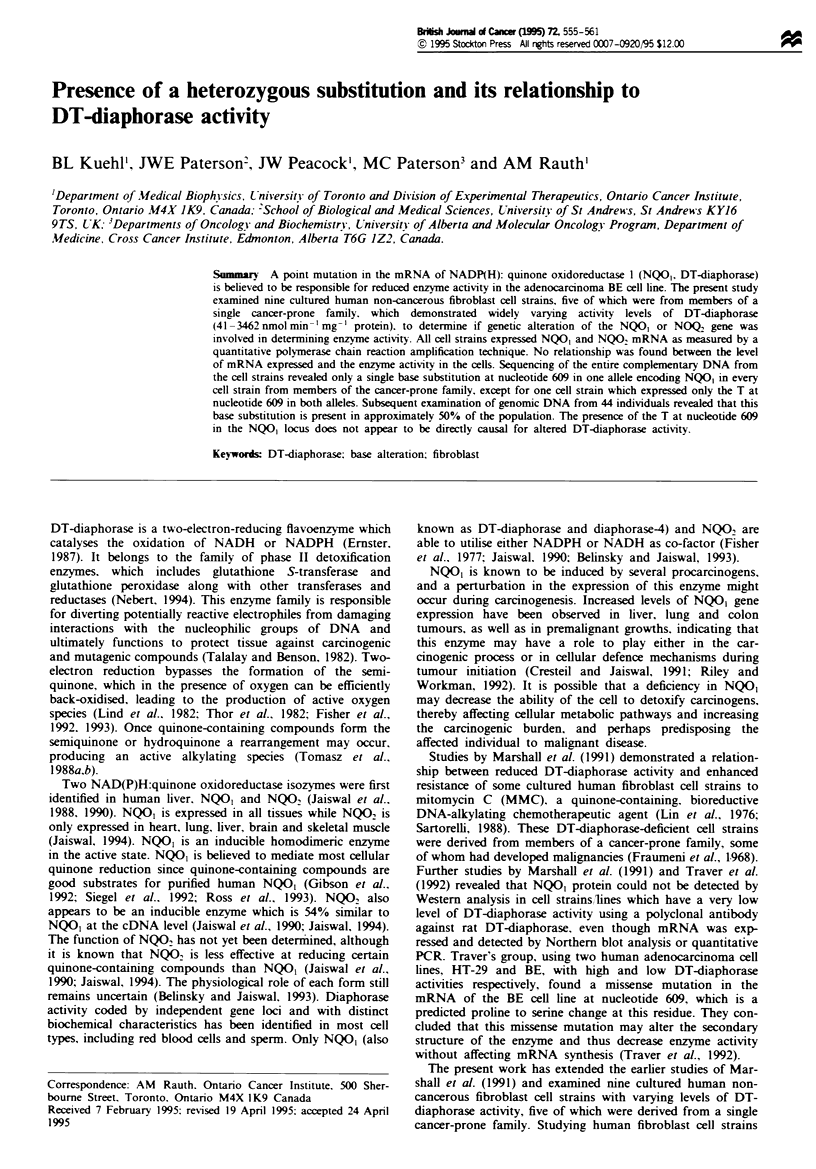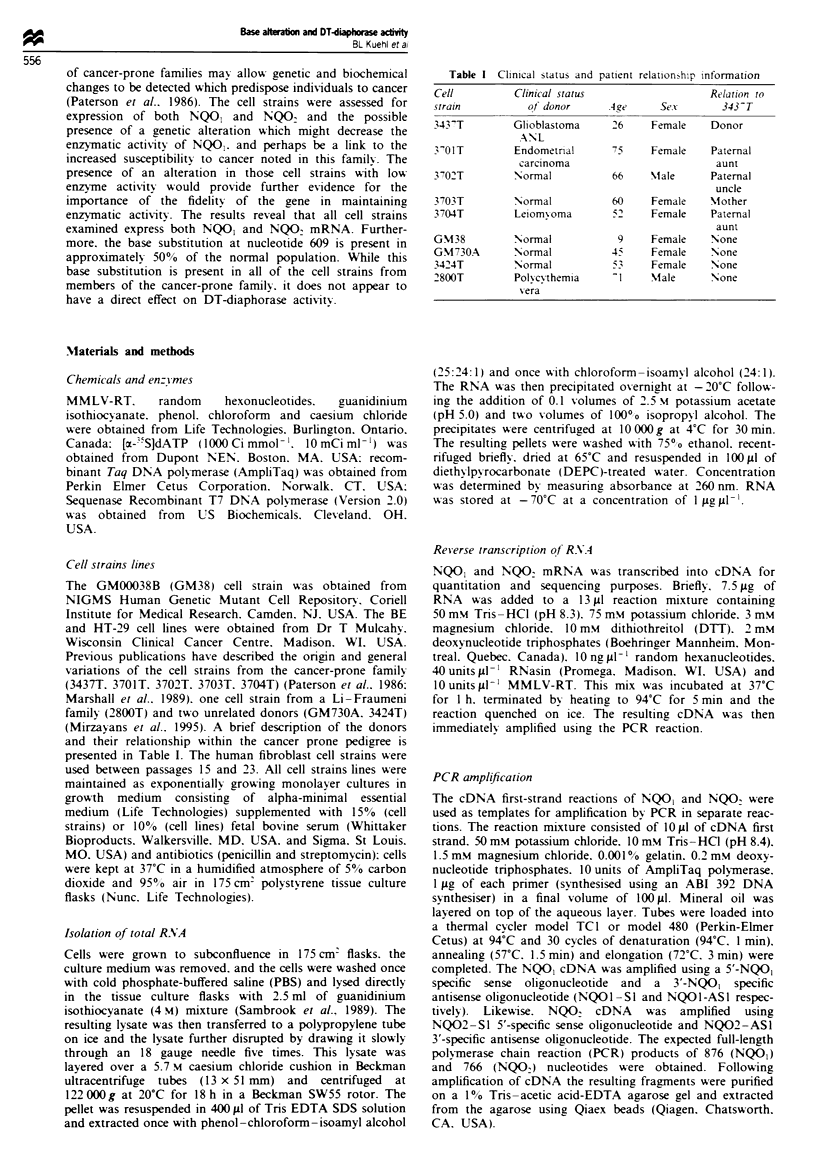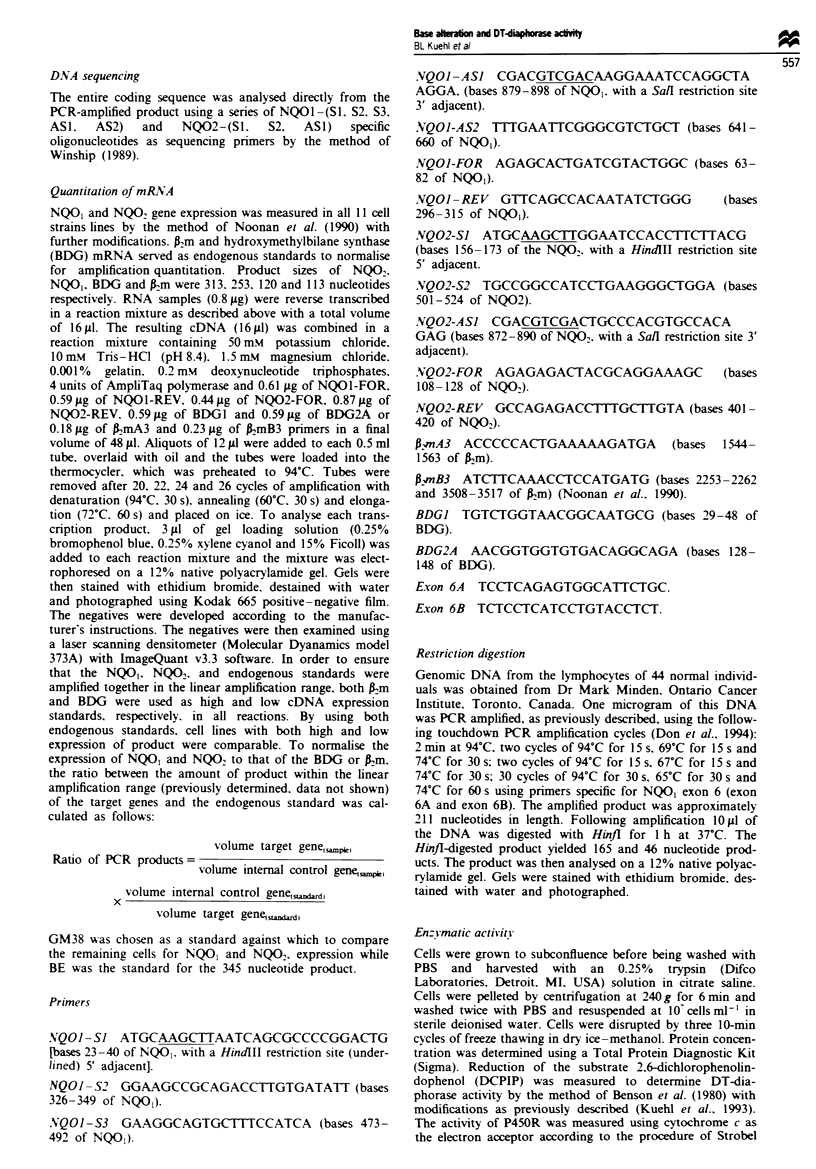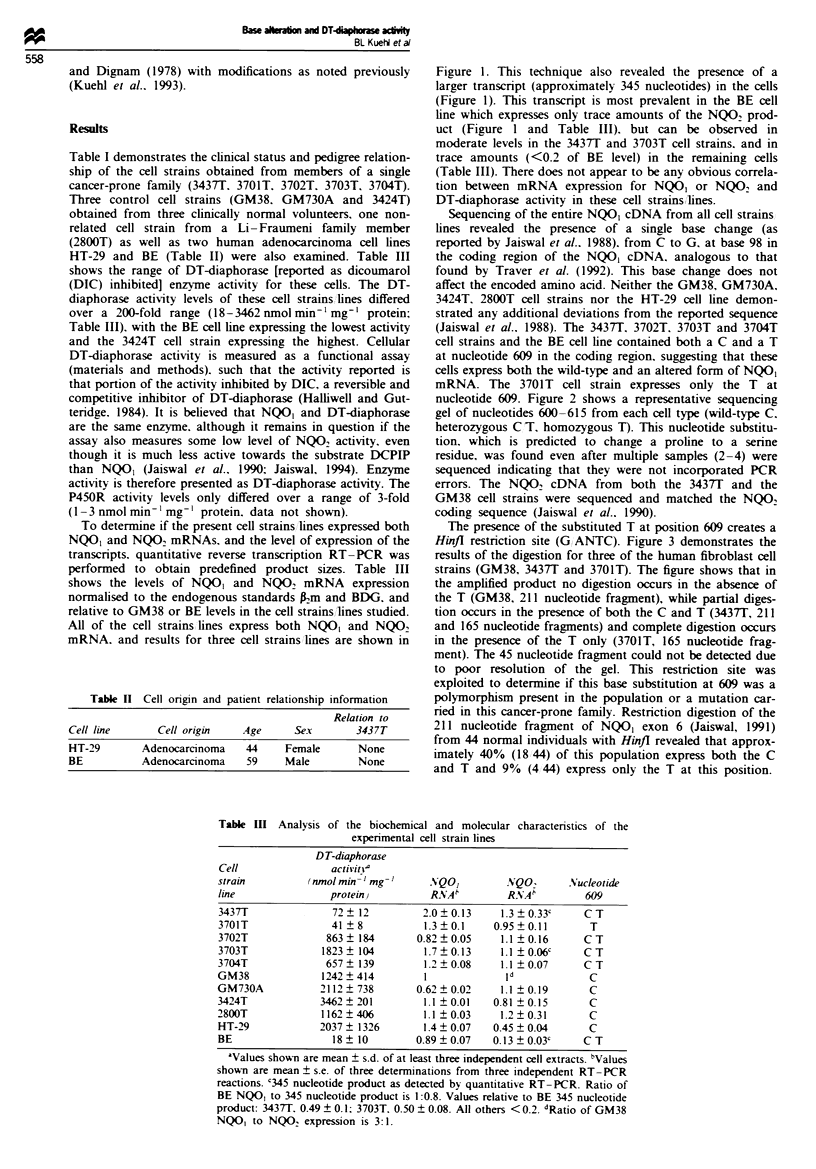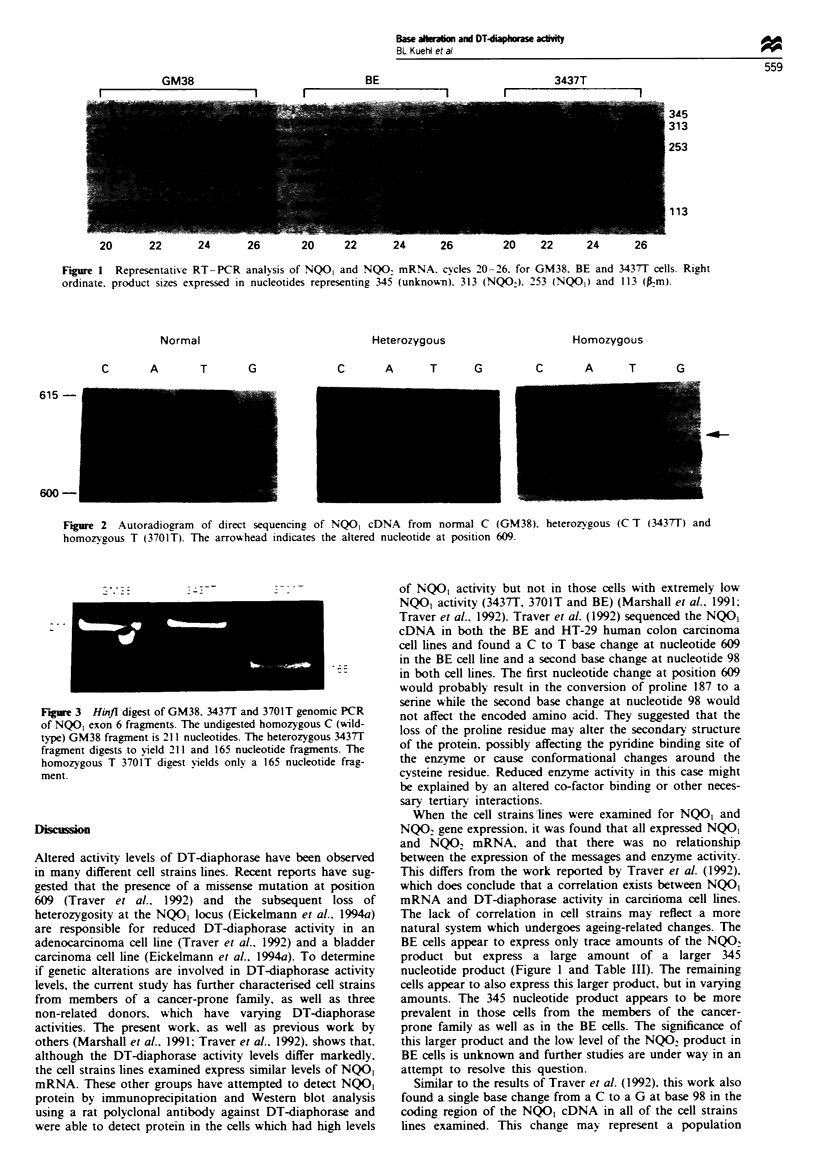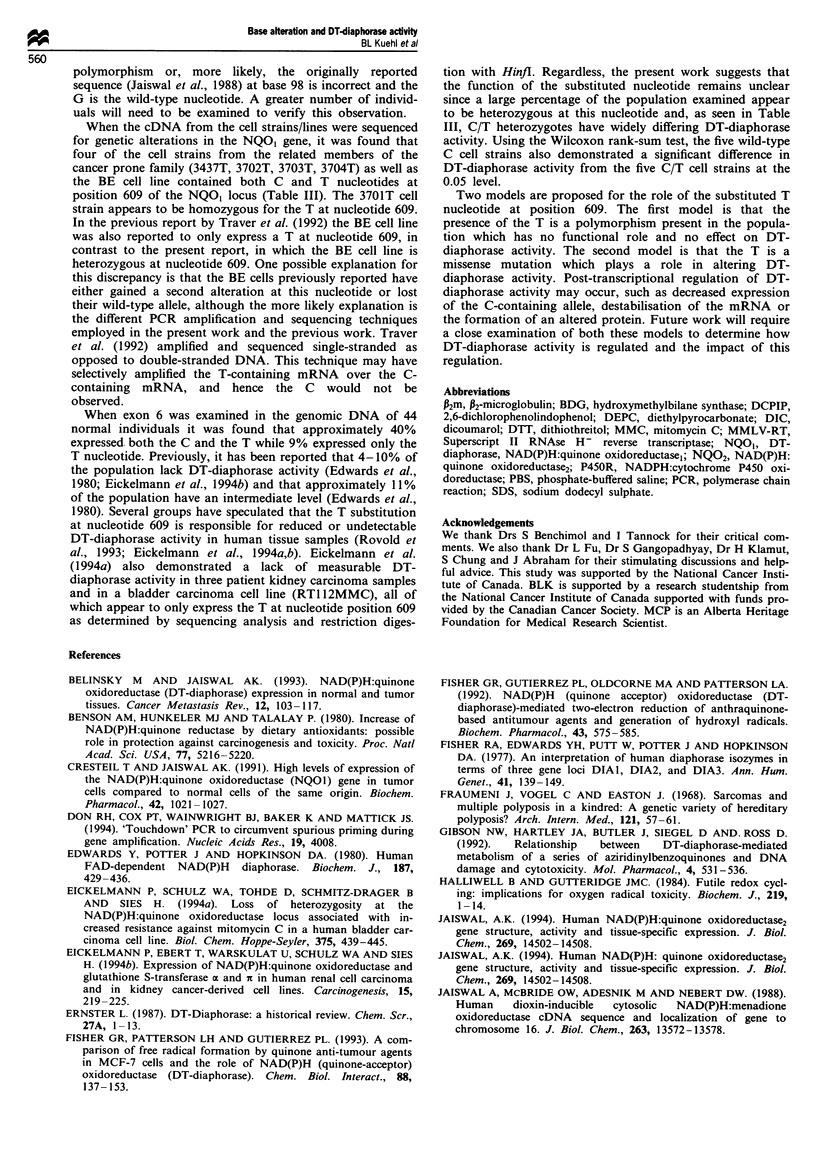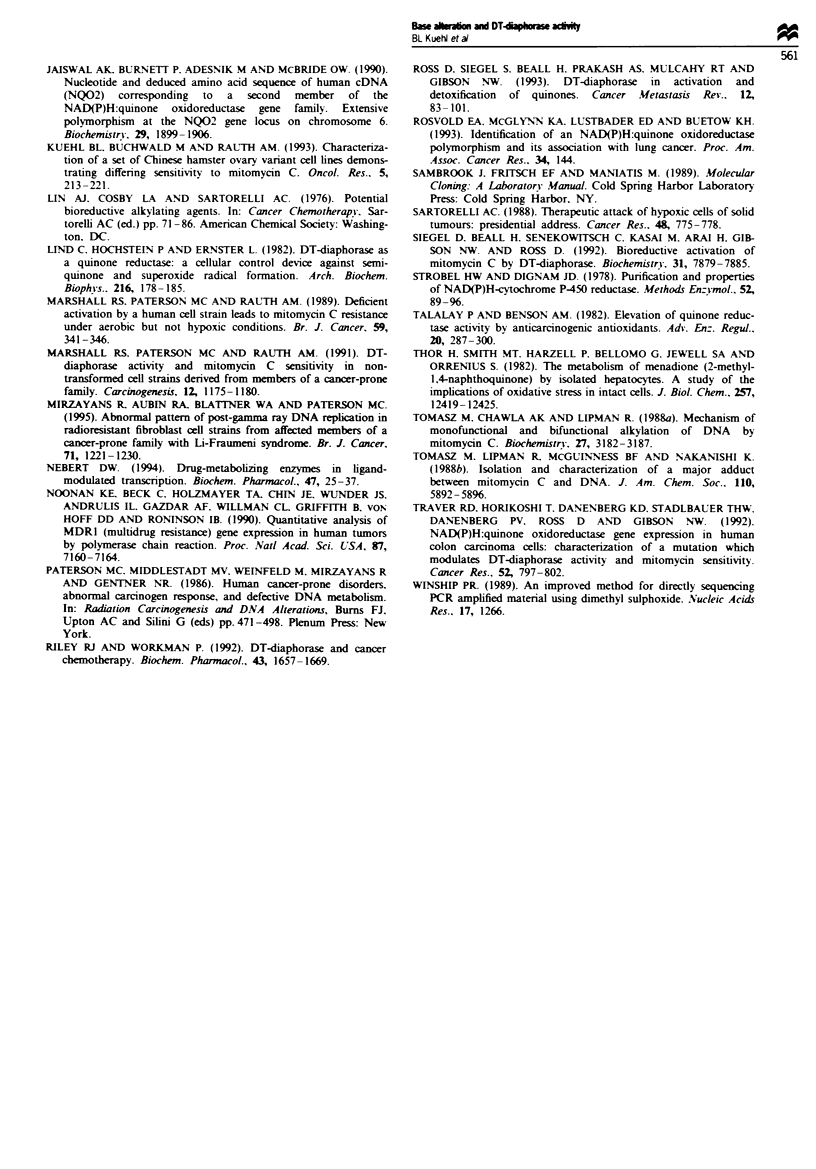# Presence of a heterozygous substitution and its relationship to DT-diaphorase activity.

**DOI:** 10.1038/bjc.1995.373

**Published:** 1995-09

**Authors:** B. L. Kuehl, J. W. Paterson, J. W. Peacock, M. C. Paterson, A. M. Rauth

**Affiliations:** Department of Medical Biophysics, University of Toronto, Ontario, Canada.

## Abstract

**Images:**


British Journal of Cancer (1995) 72, 555-561

© 1995 Stockton Press All rights reserved 0007-0920/95 $12.00

### Presence of a heterozygous substitution and its relationship to DT-diaphorase activity

BL Kuehl[1], JWE Paterson[2], JW Peacock[1], MC Paterson[3] and AM Rauth[1]

[1]*Department of Medical Biophysics, University of Toronto and Division of Experimental Therapeutics, Ontario Cancer Institute, Toronto, Ontario M4X 1K9, Canada;* [2]*School of Biological and Medical Sciences, University of St Andrews, St Andrews KY16 9TS, UK;* [3]*Departments of Oncology and Biochemistry, University of Alberta and Molecular Oncology Program, Department of Medicine, Cross Cancer Institute, Edmonton, Alberta T6G 1Z2, Canada.*

**Summary** A point mutation in the mRNA of NADP(H): quinone oxidoreductase 1 ($NQO_1$, DT-diaphorase) is believed to be responsible for reduced enzyme activity in the adenocarcinoma BE cell line. The present study examined nine cultured human non-cancerous fibroblast cell strains, five of which were from members of a single cancer-prone family, which demonstrated widely varying activity levels of DT-diaphorase (41-3462 nmol $min^{-1}$ $mg^{-1}$ protein), to determine if genetic alteration of the $NQO_1$ or $NQO_2$ gene was involved in determining enzyme activity. All cell strains expressed $NQO_1$ and $NQO_2$ mRNA as measured by a quantitative polymerase chain reaction amplification technique. No relationship was found between the level of mRNA expressed and the enzyme activity in the cells. Sequencing of the entire complementary DNA from the cell strains revealed only a single base substitution at nucleotide 609 in one allele encoding $NQO_1$ in every cell strain from members of the cancer-prone family, except for one cell strain which expressed only the T at nucleotide 609 in both alleles. Subsequent examination of genomic DNA from 44 individuals revealed that this base substitution is present in approximately 50% of the population. The presence of the T at nucleotide 609 in the $NQO_1$ locus does not appear to be directly causal for altered DT-diaphorase activity.

**Keywords:** DT-diaphorase; base alteration; fibroblast

DT-diaphorase is a two-electron-reducing flavoenzyme which catalyses the oxidation of NADH or NADPH (Ernster, 1987). It belongs to the family of phase II detoxification enzymes, which includes glutathione *S*-transferase and glutathione peroxidase along with other transferases and reductases (Nebert, 1994). This enzyme family is responsible for diverting potentially reactive electrophiles from damaging interactions with the nucleophilic groups of DNA and ultimately functions to protect tissue against carcinogenic and mutagenic compounds (Talalay and Benson, 1982). Two-electron reduction bypasses the formation of the semiquinone, which in the presence of oxygen can be efficiently back-oxidised, leading to the production of active oxygen species (Lind *et al.*, 1982; Thor *et al.*, 1982; Fisher *et al.*, 1992, 1993). Once quinone-containing compounds form the semiquinone or hydroquinone a rearrangement may occur, producing an active alkylating species (Tomasz *et al.*, 1988*a,b*).

Two NAD(P)H:quinone oxidoreductase isozymes were first identified in human liver, $NQO_1$ and $NQO_2$ (Jaiswal *et al.*, 1988, 1990). $NQO_1$ is expressed in all tissues while $NQO_2$ is only expressed in heart, lung, liver, brain and skeletal muscle (Jaiswal, 1994). $NQO_1$ is an inducible homodimeric enzyme in the active state. $NQO_1$ is believed to mediate most cellular quinone reduction since quinone-containing compounds are good substrates for purified human $NQO_1$ (Gibson *et al.*, 1992; Siegel *et al.*, 1992; Ross *et al.*, 1993). $NQO_2$ also appears to be an inducible enzyme which is 54% similar to $NQO_1$ at the cDNA level (Jaiswal *et al.*, 1990; Jaiswal, 1994). The function of $NQO_2$ has not yet been determined, although it is known that $NQO_2$ is less effective at reducing certain quinone-containing compounds than $NQO_1$ (Jaiswal *et al.*, 1990; Jaiswal, 1994). The physiological role of each form still remains uncertain (Belinsky and Jaiswal, 1993). Diaphorase activity coded by independent gene loci and with distinct biochemical characteristics has been identified in most cell types, including red blood cells and sperm. Only $NQO_1$ (also known as DT-diaphorase and diaphorase-4) and $NQO_2$ are able to utilise either NADPH or NADH as co-factor (Fisher *et al.*, 1977; Jaiswal, 1990; Belinsky and Jaiswal, 1993).

$NQO_1$ is known to be induced by several procarcinogens, and a perturbation in the expression of this enzyme might occur during carcinogenesis. Increased levels of $NQO_1$ gene expression have been observed in liver, lung and colon tumours, as well as in premalignant growths, indicating that this enzyme may have a role to play either in the carcinogenic process or in cellular defence mechanisms during tumour initiation (Cresteil and Jaiswal, 1991; Riley and Workman, 1992). It is possible that a deficiency in $NQO_1$ may decrease the ability of the cell to detoxify carcinogens, thereby affecting cellular metabolic pathways and increasing the carcinogenic burden, and perhaps predisposing the affected individual to malignant disease.

Studies by Marshall *et al.* (1991) demonstrated a relationship between reduced DT-diaphorase activity and enhanced resistance of some cultured human fibroblast cell strains to mitomycin C (MMC), a quinone-containing, bioreductive DNA-alkylating chemotherapeutic agent (Lin *et al.*, 1976; Sartorelli, 1988). These DT-diaphorase-deficient cell strains were derived from members of a cancer-prone family, some of whom had developed malignancies (Fraumeni *et al.*, 1968). Further studies by Marshall *et al.* (1991) and Traver *et al.* (1992) revealed that $NQO_1$ protein could not be detected by Western analysis in cell strains/lines which have a very low level of DT-diaphorase activity using a polyclonal antibody against rat DT-diaphorase, even though mRNA was expressed and detected by Northern blot analysis or quantitative PCR. Traver's group, using two human adenocarcinoma cell lines, HT-29 and BE, with high and low DT-diaphorase activities respectively, found a missense mutation in the mRNA of the BE cell line at nucleotide 609, which is a predicted proline to serine change at this residue. They concluded that this missense mutation may alter the secondary structure of the enzyme and thus decrease enzyme activity without affecting mRNA synthesis (Traver *et al.*, 1992).

The present work has extended the earlier studies of Marshall *et al.* (1991) and examined nine cultured human non-cancerous fibroblast cell strains with varying levels of DT-diaphorase activity, five of which were derived from a single cancer-prone family. Studying human fibroblast cell strains

Correspondence: AM Rauth, Ontario Cancer Institute, 500 Sherbourne Street, Toronto, Ontario M4X 1K9 Canada
Received 7 February 1995; revised 19 April 1995; accepted 24 April 1995

of cancer-prone families may allow genetic and biochemical changes to be detected which predispose individuals to cancer (Paterson *et al.*, 1986). The cell strains were assessed for expression of both $NQO_1$ and $NQO_2$ and the possible presence of a genetic alteration which might decrease the enzymatic activity of $NQO_1$, and perhaps be a link to the increased susceptibility to cancer noted in this family. The presence of an alteration in those cell strains with low enzyme activity would provide further evidence for the importance of the fidelity of the gene in maintaining enzymatic activity. The results reveal that all cell strains examined express both $NQO_1$ and $NQO_2$ mRNA. Furthermore, the base substitution at nucleotide 609 is present in approximately 50% of the normal population. While this base substitution is present in all of the cell strains from members of the cancer-prone family, it does not appear to have a direct effect on DT-diaphorase activity.

**Table I** Clinical status and patient relationship information

| *Cell strain* | *Clinical status of donor* | *Age* | *Sex* | *Relation to 3437T* |
|---|---|---|---|---|
| 3437T | Glioblastoma ANL | 26 | Female | Donor |
| 3701T | Endometrial carcinoma | 75 | Female | Paternal aunt |
| 3702T | Normal | 66 | Male | Paternal uncle |
| 3703T | Normal | 60 | Female | Mother |
| 3704T | Leiomyoma | 52 | Female | Paternal aunt |
| GM38 | Normal | 9 | Female | None |
| GM730A | Normal | 45 | Female | None |
| 3424T | Normal | 53 | Female | None |
| 2800T | Polycythemia vera | 71 | Male | None |

### Materials and methods

### *Chemicals and enzymes*

MMLV-RT, random hexonucleotides, guanidinium isothiocyanate, phenol, chloroform and caesium chloride were obtained from Life Technologies, Burlington, Ontario, Canada; [$\alpha$-$^{35}$S]dATP (1000 Ci mmol$^{-1}$, 10 mCi ml$^{-1}$) was obtained from Dupont NEN, Boston, MA, USA; recombinant *Taq* DNA polymerase (AmpliTaq) was obtained from Perkin Elmer Cetus Corporation, Norwalk, CT, USA; Sequenase Recombinant T7 DNA polymerase (Version 2.0) was obtained from US Biochemicals, Cleveland, OH, USA.

### *Cell strains lines*

The GM00038B (GM38) cell strain was obtained from NIGMS Human Genetic Mutant Cell Repository, Coriell Institute for Medical Research, Camden, NJ, USA. The BE and HT-29 cell lines were obtained from Dr T Mulcahy, Wisconsin Clinical Cancer Centre, Madison, WI, USA. Previous publications have described the origin and general variations of the cell strains from the cancer-prone family (3437T, 3701T, 3702T, 3703T, 3704T) (Paterson *et al.*, 1986; Marshall *et al.*, 1989), one cell strain from a Li–Fraumeni family (2800T) and two unrelated donors (GM730A, 3424T) (Mirzayans *et al.*, 1995). A brief description of the donors and their relationship within the cancer prone pedigree is presented in Table I. The human fibroblast cell strains were used between passages 15 and 23. All cell strains lines were maintained as exponentially growing monolayer cultures in growth medium consisting of alpha-minimal essential medium (Life Technologies) supplemented with 15% (cell strains) or 10% (cell lines) fetal bovine serum (Whittaker Bioproducts, Walkersville, MD, USA, and Sigma, St Louis, MO, USA) and antibiotics (penicillin and streptomycin); cells were kept at 37°C in a humidified atmosphere of 5% carbon dioxide and 95% air in 175 cm$^2$ polystyrene tissue culture flasks (Nunc, Life Technologies).

### *Isolation of total RNA*

Cells were grown to subconfluence in 175 cm$^2$ flasks, the culture medium was removed, and the cells were washed once with cold phosphate-buffered saline (PBS) and lysed directly in the tissue culture flasks with 2.5 ml of guanidinium isothiocyanate (4 M) mixture (Sambrook *et al.*, 1989). The resulting lysate was then transferred to a polypropylene tube on ice and the lysate further disrupted by drawing it slowly through an 18 gauge needle five times. This lysate was layered over a 5.7 M caesium chloride cushion in Beckman ultracentrifuge tubes (13 × 51 mm) and centrifuged at 122 000 *g* at 20°C for 18 h in a Beckman SW55 rotor. The pellet was resuspended in 400 μl of Tris EDTA SDS solution and extracted once with phenol–chloroform–isoamyl alcohol (25:24:1) and once with chloroform–isoamyl alcohol (24:1). The RNA was then precipitated overnight at −20°C following the addition of 0.1 volumes of 2.5 M potassium acetate (pH 5.0) and two volumes of 100% isopropyl alcohol. The precipitates were centrifuged at 10 000 *g* at 4°C for 30 min. The resulting pellets were washed with 75% ethanol, recentrifuged briefly, dried at 65°C and resuspended in 100 μl of diethylpyrocarbonate (DEPC)-treated water. Concentration was determined by measuring absorbance at 260 nm. RNA was stored at −70°C at a concentration of 1 μg μl$^{-1}$.

### *Reverse transcription of RNA*

$NQO_1$ and $NQO_2$ mRNA was transcribed into cDNA for quantitation and sequencing purposes. Briefly, 7.5 μg of RNA was added to a 13 μl reaction mixture containing 50 mM Tris–HCl (pH 8.3), 75 mM potassium chloride, 3 mM magnesium chloride, 10 mM dithiothreitol (DTT), 2 mM deoxynucleotide triphosphates (Boehringer Mannheim, Montreal, Quebec, Canada), 10 ng μl$^{-1}$ random hexanucleotides, 40 units μl$^{-1}$ RNasin (Promega, Madison, WI, USA) and 10 units μl$^{-1}$ MMLV-RT. This mix was incubated at 37°C for 1 h, terminated by heating to 94°C for 5 min and the reaction quenched on ice. The resulting cDNA was then immediately amplified using the PCR reaction.

### *PCR amplification*

The cDNA first-strand reactions of $NQO_1$ and $NQO_2$ were used as templates for amplification by PCR in separate reactions. The reaction mixture consisted of 10 μl of cDNA first strand, 50 mM potassium chloride, 10 mM Tris–HCl (pH 8.4), 1.5 mM magnesium chloride, 0.001% gelatin, 0.2 mM deoxynucleotide triphosphates, 10 units of AmpliTaq polymerase, 1 μg of each primer (synthesised using an ABI 392 DNA synthesiser) in a final volume of 100 μl. Mineral oil was layered on top of the aqueous layer. Tubes were loaded into a thermal cycler model TC1 or model 480 (Perkin-Elmer Cetus) at 94°C and 30 cycles of denaturation (94°C, 1 min), annealing (57°C, 1.5 min) and elongation (72°C, 3 min) were completed. The $NQO_1$ cDNA was amplified using a 5′-$NQO_1$ specific sense oligonucleotide and a 3′-$NQO_1$ specific antisense oligonucleotide (NQO1–S1 and NQO1-AS1 respectively). Likewise, $NQO_2$ cDNA was amplified using NQO2–S1 5′-specific sense oligonucleotide and NQO2–AS1 3′-specific antisense oligonucleotide. The expected full-length polymerase chain reaction (PCR) products of 876 ($NQO_1$) and 766 ($NQO_2$) nucleotides were obtained. Following amplification of cDNA the resulting fragments were purified on a 1% Tris–acetic acid-EDTA agarose gel and extracted from the agarose using Qiaex beads (Qiagen, Chatsworth, CA, USA).

*DNA sequencing*

The entire coding sequence was analysed directly from the PCR-amplified product using a series of NQO1-(S1, S2, S3, AS1, AS2) and NQO2-(S1, S2, AS1) specific oligonucleotides as sequencing primers by the method of Winship (1989).

*Quantitation of mRNA*

$NQO_1$ and $NQO_2$ gene expression was measured in all 11 cell strains lines by the method of Noonan *et al.* (1990) with further modifications. $\beta_2$m and hydroxymethylbilane synthase (BDG) mRNA served as endogenous standards to normalise for amplification quantitation. Product sizes of $NQO_2$, $NQO_1$, BDG and $\beta_2$m were 313, 253, 120 and 113 nucleotides respectively. RNA samples (0.8 μg) were reverse transcribed in a reaction mixture as described above with a total volume of 16 μl. The resulting cDNA (16 μl) was combined in a reaction mixture containing 50 mM potassium chloride, 10 mM Tris-HCl (pH 8.4), 1.5 mM magnesium chloride, 0.001% gelatin, 0.2 mM deoxynucleotide triphosphates, 4 units of AmpliTaq polymerase and 0.61 μg of NQO1-FOR, 0.59 μg of NQO1-REV, 0.44 μg of NQO2-FOR, 0.87 μg of NQO2-REV, 0.59 μg of BDG1 and 0.59 μg of BDG2A or 0.18 μg of $\beta_2$mA3 and 0.23 μg of $\beta_2$mB3 primers in a final volume of 48 μl. Aliquots of 12 μl were added to each 0.5 ml tube, overlaid with oil and the tubes were loaded into the thermocycler, which was preheated to 94°C. Tubes were removed after 20, 22, 24 and 26 cycles of amplification with denaturation (94°C, 30 s), annealing (60°C, 30 s) and elongation (72°C, 60 s) and placed on ice. To analyse each transcription product, 3 μl of gel loading solution (0.25% bromophenol blue, 0.25% xylene cyanol and 15% Ficoll) was added to each reaction mixture and the mixture was electrophoresed on a 12% native polyacrylamide gel. Gels were then stained with ethidium bromide, destained with water and photographed using Kodak 665 positive-negative film. The negatives were developed according to the manufacturer's instructions. The negatives were then examined using a laser scanning densitometer (Molecular Dyanamics model 373A) with ImageQuant v3.3 software. In order to ensure that the $NQO_1$, $NQO_2$, and endogenous standards were amplified together in the linear amplification range, both $\beta_2$m and BDG were used as high and low cDNA expression standards, respectively, in all reactions. By using both endogenous standards, cell lines with both high and low expression of product were comparable. To normalise the expression of $NQO_1$ and $NQO_2$ to that of the BDG or $\beta_2$m, the ratio between the amount of product within the linear amplification range (previously determined, data not shown) of the target genes and the endogenous standard was calculated as follows:

$$\text{Ratio of PCR products} = \frac{\text{volume target gene}_{(\text{sample})}}{\text{volume internal control gene}_{(\text{sample})}} \times \frac{\text{volume internal control gene}_{(\text{standard})}}{\text{volume target gene}_{(\text{standard})}}$$

GM38 was chosen as a standard against which to compare the remaining cells for $NQO_1$ and $NQO_2$, expression while BE was the standard for the 345 nucleotide product.

*Primers*

*NQO1-S1* ATGCAAGCTTAATCAGCGCCCCGGACTG [bases 23-40 of $NQO_1$, with a *Hin*dIII restriction site (underlined) 5′ adjacent].

*NQO1-S2* GGAAGCCGCAGACCTTGTGATATT (bases 326-349 of $NQO_1$).

*NQO1-S3* GAAGGCAGTGCTTTCCATCA (bases 473-492 of $NQO_1$).

*NQO1-AS1* CGACGTCGACAAGGAAATCCAGGCTA AGGA, (bases 879-898 of $NQO_1$, with a *Sal*I restriction site 3′ adjacent).

*NQO1-AS2* TTTGAATTCGGGCGTCTGCT (bases 641-660 of $NQO_1$).

*NQO1-FOR* AGAGCACTGATCGTACTGGC (bases 63-82 of $NQO_1$).

*NQO1-REV* GTTCAGCCACAATATCTGGG (bases 296-315 of $NQO_1$).

*NQO2-S1* ATGCAAGCTTGGAATCCACCTTCTTACG (bases 156-173 of the $NQO_2$, with a *Hin*dIII restriction site 5′ adjacent.

*NQO2-S2* TGCCGGCCATCCTGAAGGGCTGGA (bases 501-524 of NQO2).

*NQO2-AS1* CGACGTCGACTGCCCACGTGCCACA GAG (bases 872-890 of $NQO_2$, with a *Sal*I restriction site 3′ adjacent).

*NQO2-FOR* AGAGAGACTACGCAGGAAAGC (bases 108-128 of $NQO_2$).

*NQO2-REV* GCCAGAGACCTTTGCTTGTA (bases 401-420 of $NQO_2$).

*$\beta_2$mA3* ACCCCCACTGAAAAAGATGA (bases 1544-1563 of $\beta_2$m).

*$\beta_2$mB3* ATCTTCAAACCTCCATGATG (bases 2253-2262 and 3508-3517 of $\beta_2$m) (Noonan *et al.*, 1990).

*BDG1* TGTCTGGTAACGGCAATGCG (bases 29-48 of BDG).

*BDG2A* AACGGTGGTGTGACAGGCAGA (bases 128-148 of BDG).

*Exon 6A* TCCTCAGAGTGGCATTCTGC.

*Exon 6B* TCTCCTCATCCTGTACCTCT.

*Restriction digestion*

Genomic DNA from the lymphocytes of 44 normal individuals was obtained from Dr Mark Minden, Ontario Cancer Institute, Toronto, Canada. One microgram of this DNA was PCR amplified, as previously described, using the following touchdown PCR amplification cycles (Don *et al.*, 1994): 2 min at 94°C, two cycles of 94°C for 15 s, 69°C for 15 s and 74°C for 30 s; two cycles of 94°C for 15 s, 67°C for 15 s and 74°C for 30 s; 30 cycles of 94°C for 30 s, 65°C for 30 s and 74°C for 60 s using primers specific for $NQO_1$ exon 6 (exon 6A and exon 6B). The amplified product was approximately 211 nucleotides in length. Following amplification 10 μl of the DNA was digested with *Hin*fI for 1 h at 37°C. The *Hin*fI-digested product yielded 165 and 46 nucleotide products. The product was then analysed on a 12% native polyacrylamide gel. Gels were stained with ethidium bromide, destained with water and photographed.

*Enzymatic activity*

Cells were grown to subconfluence before being washed with PBS and harvested with an 0.25% trypsin (Difco Laboratories, Detroit, MI, USA) solution in citrate saline. Cells were pelleted by centrifugation at 240 *g* for 6 min and washed twice with PBS and resuspended at $10^7$ cells $ml^{-1}$ in sterile deionised water. Cells were disrupted by three 10-min cycles of freeze/thawing in dry ice-methanol. Protein concentration was determined using a Total Protein Diagnostic Kit (Sigma). Reduction of the substrate 2,6-dichlorophenolindophenol (DCPIP) was measured to determine DT-diaphorase activity by the method of Benson *et al.* (1980) with modifications as previously described (Kuehl *et al.*, 1993). The activity of P450R was measured using cytochrome *c* as the electron acceptor according to the procedure of Strobel and Dignam (1978) with modifications as noted previously (Kuehl et al., 1993).

### Results

Table I demonstrates the clinical status and pedigree relationship of the cell strains obtained from members of a single cancer-prone family (3437T, 3701T, 3702T, 3703T, 3704T). Three control cell strains (GM38, GM730A and 3424T) obtained from three clinically normal volunteers, one non-related cell strain from a Li–Fraumeni family member (2800T) as well as two human adenocarcinoma cell lines HT-29 and BE (Table II) were also examined. Table III shows the range of DT-diaphorase [reported as dicoumarol (DIC) inhibited] enzyme activity for these cells. The DT-diaphorase activity levels of these cell strains/lines differed over a 200-fold range (18–3462 nmol $min^{-1}$ $mg^{-1}$ protein; Table III), with the BE cell line expressing the lowest activity and the 3424T cell strain expressing the highest. Cellular DT-diaphorase activity is measured as a functional assay (materials and methods), such that the activity reported is that portion of the activity inhibited by DIC, a reversible and competitive inhibitor of DT-diaphorase (Halliwell and Gutteridge, 1984). It is believed that $NQO_1$ and DT-diaphorase are the same enzyme, although it remains in question if the assay also measures some low level of $NQO_2$ activity, even though it is much less active towards the substrate DCPIP than $NQO_1$ (Jaiswal et al., 1990; Jaiswal, 1994). Enzyme activity is therefore presented as DT-diaphorase activity. The P450R activity levels only differed over a range of 3-fold (1–3 nmol $min^{-1}$ $mg^{-1}$ protein, data not shown).

To determine if the present cell strains/lines expressed both $NQO_1$ and $NQO_2$ mRNAs, and the level of expression of the transcripts, quantitative reverse transcription RT–PCR was performed to obtain predefined product sizes. Table III shows the levels of $NQO_1$ and $NQO_2$ mRNA expression normalised to the endogenous standards $\beta_2$m and BDG, and relative to GM38 or BE levels in the cell strains/lines studied. All of the cell strains/lines express both $NQO_1$ and $NQO_2$ mRNA, and results for three cell strains/lines are shown in Figure 1. This technique also revealed the presence of a larger transcript (approximately 345 nucleotides) in the cells (Figure 1). This transcript is most prevalent in the BE cell line which expresses only trace amounts of the $NQO_2$ product (Figure 1 and Table III), but can be observed in moderate levels in the 3437T and 3703T cell strains, and in trace amounts (<0.2 of BE level) in the remaining cells (Table III). There does not appear to be any obvious correlation between mRNA expression for $NQO_1$ or $NQO_2$ and DT-diaphorase activity in these cell strains/lines.

Sequencing of the entire $NQO_1$ cDNA from all cell strains/lines revealed the presence of a single base change (as reported by Jaiswal et al., 1988), from C to G, at base 98 in the coding region of the $NQO_1$ cDNA, analogous to that found by Traver et al. (1992). This base change does not affect the encoded amino acid. Neither the GM38, GM730A, 3424T, 2800T cell strains nor the HT-29 cell line demonstrated any additional deviations from the reported sequence (Jaiswal et al., 1988). The 3437T, 3702T, 3703T and 3704T cell strains and the BE cell line contained both a C and a T at nucleotide 609 in the coding region, suggesting that these cells express both the wild-type and an altered form of $NQO_1$ mRNA. The 3701T cell strain expresses only the T at nucleotide 609. Figure 2 shows a representative sequencing gel of nucleotides 600–615 from each cell type (wild-type C, heterozygous C/T, homozygous T). This nucleotide substitution, which is predicted to change a proline to a serine residue, was found even after multiple samples (2–4) were sequenced indicating that they were not incorporated PCR errors. The $NQO_2$ cDNA from both the 3437T and the GM38 cell strains were sequenced and matched the $NQO_2$ coding sequence (Jaiswal et al., 1990).

The presence of the substituted T at position 609 creates a *Hinf*I restriction site (G/ANTC). Figure 3 demonstrates the results of the digestion for three of the human fibroblast cell strains (GM38, 3437T and 3701T). The figure shows that in the amplified product no digestion occurs in the absence of the T (GM38, 211 nucleotide fragment), while partial digestion occurs in the presence of both the C and T (3437T, 211 and 165 nucleotide fragments) and complete digestion occurs in the presence of the T only (3701T, 165 nucleotide fragment). The 45 nucleotide fragment could not be detected due to poor resolution of the gel. This restriction site was exploited to determine if this base substitution at 609 was a polymorphism present in the population or a mutation carried in this cancer-prone family. Restriction digestion of the 211 nucleotide fragment of $NQO_1$ exon 6 (Jaiswal, 1991) from 44 normal individuals with *Hinf*I revealed that approximately 40% (18/44) of this population express both the C and T and 9% (4/44) express only the T at this position.

**Table II** Cell origin and patient relationship information

| Cell line | Cell origin | Age | Sex | Relation to 3437T |
|---|---|---|---|---|
| HT-29 | Adenocarcinoma | 44 | Female | None |
| BE | Adenocarcinoma | 59 | Male | None |

**Table III** Analysis of the biochemical and molecular characteristics of the experimental cell strain lines

| Cell strain/line | DT-diaphorase activity[a] (nmol $min^{-1}$ $mg^{-1}$ protein) | $NQO_1$ RNA[b] | $NQO_2$ RNA[b] | Nucleotide 609 |
|---|---|---|---|---|
| 3437T | 72 ± 12 | 2.0 ± 0.13 | 1.3 ± 0.33[c] | C T |
| 3701T | 41 ± 8 | 1.3 ± 0.1 | 0.95 ± 0.11 | T |
| 3702T | 863 ± 184 | 0.82 ± 0.05 | 1.1 ± 0.16 | C T |
| 3703T | 1823 ± 104 | 1.7 ± 0.13 | 1.1 ± 0.06[c] | C T |
| 3704T | 657 ± 139 | 1.2 ± 0.08 | 1.1 ± 0.07 | C T |
| GM38 | 1242 ± 414 | 1 | 1[d] | C |
| GM730A | 2112 ± 738 | 0.62 ± 0.02 | 1.1 ± 0.19 | C |
| 3424T | 3462 ± 201 | 1.1 ± 0.01 | 0.81 ± 0.15 | C |
| 2800T | 1162 ± 406 | 1.1 ± 0.03 | 1.2 ± 0.31 | C |
| HT-29 | 2037 ± 1326 | 1.4 ± 0.07 | 0.45 ± 0.04 | C |
| BE | 18 ± 10 | 0.89 ± 0.07 | 0.13 ± 0.03[c] | C T |

[a]Values shown are mean ± s.d. of at least three independent cell extracts. [b]Values shown are mean ± s.e. of three determinations from three independent RT–PCR reactions. [c]345 nucleotide product as detected by quantitative RT–PCR. Ratio of BE $NQO_1$ to 345 nucleotide product is 1:0.8. Values relative to BE 345 nucleotide product: 3437T, 0.49 ± 0.1; 3703T, 0.50 ± 0.08. All others <0.2. [d]Ratio of GM38 $NQO_1$ to $NQO_2$ expression is 3:1.

**Figure 1** Representative RT-PCR analysis of $NQO_1$ and $NQO_2$ mRNA, cycles 20–26, for GM38, BE and 3437T cells. Right ordinate, product sizes expressed in nucleotides representing 345 (unknown), 313 ($NQO_2$), 253 ($NQO_1$) and 113 ($\beta_2$m).

**Figure 2** Autoradiogram of direct sequencing of $NQO_1$ cDNA from normal C (GM38), heterozygous (C T (3437T) and homozygous T (3701T). The arrowhead indicates the altered nucleotide at position 609.

**Figure 3** *HinfI* digest of GM38, 3437T and 3701T genomic PCR of $NQO_1$ exon 6 fragments. The undigested homozygous C (wild-type) GM38 fragment is 211 nucleotides. The heterozygous 3437T fragment digests to yield 211 and 165 nucleotide fragments. The homozygous T 3701T digest yields only a 165 nucleotide fragment.

### Discussion

Altered activity levels of DT-diaphorase have been observed in many different cell strains lines. Recent reports have suggested that the presence of a missense mutation at position 609 (Traver *et al.*, 1992) and the subsequent loss of heterozygosity at the $NQO_1$ locus (Eickelmann *et al.*, 1994*a*) are responsible for reduced DT-diaphorase activity in an adenocarcinoma cell line (Traver *et al.*, 1992) and a bladder carcinoma cell line (Eickelmann *et al.*, 1994*a*). To determine if genetic alterations are involved in DT-diaphorase activity levels, the current study has further characterised cell strains from members of a cancer-prone family, as well as three non-related donors, which have varying DT-diaphorase activities. The present work, as well as previous work by others (Marshall *et al.*, 1991; Traver *et al.*, 1992), shows that, although the DT-diaphorase activity levels differ markedly, the cell strains lines examined express similar levels of $NQO_1$ mRNA. These other groups have attempted to detect $NQO_1$ protein by immunoprecipitation and Western blot analysis using a rat polyclonal antibody against DT-diaphorase and were able to detect protein in the cells which had high levels of $NQO_1$ activity but not in those cells with extremely low $NQO_1$ activity (3437T, 3701T and BE) (Marshall *et al.*, 1991; Traver *et al.*, 1992). Traver *et al.* (1992) sequenced the $NQO_1$ cDNA in both the BE and HT-29 human colon carcinoma cell lines and found a C to T base change at nucleotide 609 in the BE cell line and a second base change at nucleotide 98 in both cell lines. The first nucleotide change at position 609 would probably result in the conversion of proline 187 to a serine while the second base change at nucleotide 98 would not affect the encoded amino acid. They suggested that the loss of the proline residue may alter the secondary structure of the protein, possibly affecting the pyridine binding site of the enzyme or cause conformational changes around the cysteine residue. Reduced enzyme activity in this case might be explained by an altered co-factor binding or other necessary tertiary interactions.

When the cell strains lines were examined for $NQO_1$ and $NQO_2$ gene expression, it was found that all expressed $NQO_1$ and $NQO_2$ mRNA, and that there was no relationship between the expression of the messages and enzyme activity. This differs from the work reported by Traver *et al.* (1992), which does conclude that a correlation exists between $NQO_1$ mRNA and DT-diaphorase activity in carcinoma cell lines. The lack of correlation in cell strains may reflect a more natural system which undergoes ageing-related changes. The BE cells appear to express only trace amounts of the $NQO_2$ product but express a large amount of a larger 345 nucleotide product (Figure 1 and Table III). The remaining cells appear to also express this larger product, but in varying amounts. The 345 nucleotide product appears to be more prevalent in those cells from the members of the cancer-prone family as well as in the BE cells. The significance of this larger product and the low level of the $NQO_2$ product in BE cells is unknown and further studies are under way in an attempt to resolve this question.

Similar to the results of Traver *et al.* (1992), this work also found a single base change from a C to a G at base 98 in the coding region of the $NQO_1$ cDNA in all of the cell strains lines examined. This change may represent a population polymorphism or, more likely, the originally reported sequence (Jaiswal et al., 1988) at base 98 is incorrect and the G is the wild-type nucleotide. A greater number of individuals will need to be examined to verify this observation.

When the cDNA from the cell strains/lines were sequenced for genetic alterations in the $NQO_1$ gene, it was found that four of the cell strains from the related members of the cancer prone family (3437T, 3702T, 3703T, 3704T) as well as the BE cell line contained both C and T nucleotides at position 609 of the $NQO_1$ locus (Table III). The 3701T cell strain appears to be homozygous for the T at nucleotide 609. In the previous report by Traver et al. (1992) the BE cell line was also reported to only express a T at nucleotide 609, in contrast to the present report, in which the BE cell line is heterozygous at nucleotide 609. One possible explanation for this discrepancy is that the BE cells previously reported have either gained a second alteration at this nucleotide or lost their wild-type allele, although the more likely explanation is the different PCR amplification and sequencing techniques employed in the present work and the previous work. Traver et al. (1992) amplified and sequenced single-stranded as opposed to double-stranded DNA. This technique may have selectively amplified the T-containing mRNA over the C-containing mRNA, and hence the C would not be observed.

When exon 6 was examined in the genomic DNA of 44 normal individuals it was found that approximately 40% expressed both the C and the T while 9% expressed only the T nucleotide. Previously, it has been reported that 4–10% of the population lack DT-diaphorase activity (Edwards et al., 1980; Eickelmann et al., 1994b) and that approximately 11% of the population have an intermediate level (Edwards et al., 1980). Several groups have speculated that the T substitution at nucleotide 609 is responsible for reduced or undetectable DT-diaphorase activity in human tissue samples (Rovold et al., 1993; Eickelmann et al., 1994a,b). Eickelmann et al. (1994a) also demonstrated a lack of measurable DT-diaphorase activity in three patient kidney carcinoma samples and in a bladder carcinoma cell line (RT112MMC), all of which appear to only express the T at nucleotide position 609 as determined by sequencing analysis and restriction digestion with HinfI. Regardless, the present work suggests that the function of the substituted nucleotide remains unclear since a large percentage of the population examined appear to be heterozygous at this nucleotide and, as seen in Table III, C/T heterozygotes have widely differing DT-diaphorase activity. Using the Wilcoxon rank-sum test, the five wild-type C cell strains also demonstrated a significant difference in DT-diaphorase activity from the five C/T cell strains at the 0.05 level.

Two models are proposed for the role of the substituted T nucleotide at position 609. The first model is that the presence of the T is a polymorphism present in the population which has no functional role and no effect on DT-diaphorase activity. The second model is that the T is a missense mutation which plays a role in altering DT-diaphorase activity. Post-transcriptional regulation of DT-diaphorase activity may occur, such as decreased expression of the C-containing allele, destabilisation of the mRNA or the formation of an altered protein. Future work will require a close examination of both these models to determine how DT-diaphorase activity is regulated and the impact of this regulation.

**Abbreviations**

$\beta_2$m, $\beta_2$-microglobulin; BDG, hydroxymethylbilane synthase; DCPIP, 2,6-dichlorophenolindophenol; DEPC, diethylpyrocarbonate; DIC, dicoumarol; DTT, dithiothreitol; MMC, mitomycin C; MMLV-RT, Superscript II RNAse $H^-$ reverse transcriptase; $NQO_1$, DT-diaphorase, NAD(P)H:quinone $oxidoreductase_1$; $NQO_2$, NAD(P)H: quinone $oxidoreductase_2$; P450R, NADPH:cytochrome P450 oxidoreductase; PBS, phosphate-buffered saline; PCR, polymerase chain reaction; SDS, sodium dodecyl sulphate.

**Acknowledgements**

We thank Drs S Benchimol and I Tannock for their critical comments. We also thank Dr L Fu, Dr S Gangopadhyay, Dr H Klamut, S Chung and J Abraham for their stimulating discussions and helpful advice. This study was supported by the National Cancer Institute of Canada. BLK is supported by a research studentship from the National Cancer Institute of Canada supported with funds provided by the Canadian Cancer Society. MCP is an Alberta Heritage Foundation for Medical Research Scientist.

**References**

BELINSKY M AND JAISWAL AK. (1993). NAD(P)H:quinone oxidoreductase (DT-diaphorase) expression in normal and tumor tissues. *Cancer Metastasis Rev.*, **12**, 103–117.

BENSON AM, HUNKELER MJ AND TALALAY P. (1980). Increase of NAD(P)H:quinone reductase by dietary antioxidants: possible role in protection against carcinogenesis and toxicity. *Proc. Natl Acad. Sci. USA*, **77**, 5216–5220.

CRESTEIL T AND JAISWAL AK. (1991). High levels of expression of the NAD(P)H:quinone oxidoreductase (NQO1) gene in tumor cells compared to normal cells of the same origin. *Biochem. Pharmacol.*, **42**, 1021–1027.

DON RH, COX PT, WAINWRIGHT BJ, BAKER K AND MATTICK JS. (1994). 'Touchdown' PCR to circumvent spurious priming during gene amplification. *Nucleic Acids Res.*, **19**, 4008.

EDWARDS Y, POTTER J AND HOPKINSON DA. (1980). Human FAD-dependent NAD(P)H diaphorase. *Biochem. J.*, **187**, 429–436.

EICKELMANN P, SCHULZ WA, TOHDE D, SCHMITZ-DRAGER B AND SIES H. (1994a). Loss of heterozygosity at the NAD(P)H:quinone oxidoreductase locus associated with increased resistance against mitomycin C in a human bladder carcinoma cell line. *Biol. Chem. Hoppe-Seyler*, **375**, 439–445.

EICKELMANN P, EBERT T, WARSKULAT U, SCHULZ WA AND SIES H. (1994b). Expression of NAD(P)H:quinone oxidoreductase and glutathione S-transferase $\alpha$ and $\pi$ in human renal cell carcinoma and in kidney cancer-derived cell lines. *Carcinogenesis*, **15**, 219–225.

ERNSTER L. (1987). DT-Diaphorase: a historical review. *Chem. Scr.*, **27A**, 1–13.

FISHER GR, PATTERSON LH AND GUTIERREZ PL. (1993). A comparison of free radical formation by quinone anti-tumour agents in MCF-7 cells and the role of NAD(P)H (quinone-acceptor) oxidoreductase (DT-diaphorase). *Chem. Biol. Interact.*, **88**, 137–153.

FISHER GR, GUTIERREZ PL, OLDCORNE MA AND PATTERSON LA. (1992). NAD(P)H (quinone acceptor) oxidoreductase (DT-diaphorase)-mediated two-electron reduction of anthraquinone-based antitumour agents and generation of hydroxyl radicals. *Biochem. Pharmacol.*, **43**, 575–585.

FISHER RA, EDWARDS YH, PUTT W, POTTER J AND HOPKINSON DA. (1977). An interpretation of human diaphorase isozymes in terms of three gene loci DIA1, DIA2, and DIA3. *Ann. Hum. Genet.*, **41**, 139–149.

FRAUMENI J, VOGEL C AND EASTON J. (1968). Sarcomas and multiple polyposis in a kindred: A genetic variety of hereditary polyposis? *Arch. Intern. Med.*, **121**, 57–61.

GIBSON NW, HARTLEY JA, BUTLER J, SIEGEL D AND ROSS D. (1992). Relationship between DT-diaphorase-mediated metabolism of a series of aziridinylbenzoquinones and DNA damage and cytotoxicity. *Mol. Pharmacol.*, **4**, 531–536.

HALLIWELL B AND GUTTERIDGE JMC. (1984). Futile redox cycling: implications for oxygen radical toxicity. *Biochem. J.*, **219**, 1–14.

JAISWAL, A.K. (1994). Human NAD(P)H:quinone $oxidoreductase_2$ gene structure, activity and tissue-specific expression. *J. Biol. Chem.*, **269**, 14502–14508.

JAISWAL, A.K. (1994). Human NAD(P)H: quinone $oxidoreductase_2$ gene structure, activity and tissue-specific expression. *J. Biol. Chem.*, **269**, 14502–14508.

JAISWAL A, MCBRIDE OW, ADESNIK M AND NEBERT DW. (1988). Human dioxin-inducible cytosolic NAD(P)H:menadione oxidoreductase cDNA sequence and localization of gene to chromosome 16. *J. Biol. Chem.*, **263**, 13572–13578.

JAISWAL AK, BURNETT P, ADESNIK M AND MCBRIDE OW. (1990). Nucleotide and deduced amino acid sequence of human cDNA (NQO2) corresponding to a second member of the NAD(P)H:quinone oxidoreductase gene family. Extensive polymorphism at the NQO2 gene locus on chromosome 6. *Biochemistry*, **29**, 1899-1906.

KUEHL BL, BUCHWALD M AND RAUTH AM. (1993). Characterization of a set of Chinese hamster ovary variant cell lines demonstrating differing sensitivity to mitomycin C. *Oncol. Res.*, **5**, 213-221.

LIN AJ, COSBY LA AND SARTORELLI AC. (1976). Potential bioreductive alkylating agents. In: *Cancer Chemotherapy*, Sartorelli AC (ed.) pp. 71-86. American Chemical Society: Washington, DC.

LIND C, HOCHSTEIN P AND ERNSTER L. (1982). DT-diaphorase as a quinone reductase: a cellular control device against semiquinone and superoxide radical formation. *Arch. Biochem. Biophys.*, **216**, 178-185.

MARSHALL RS, PATERSON MC AND RAUTH AM. (1989). Deficient activation by a human cell strain leads to mitomycin C resistance under aerobic but not hypoxic conditions. *Br. J. Cancer*, **59**, 341-346.

MARSHALL RS, PATERSON MC AND RAUTH AM. (1991). DT-diaphorase activity and mitomycin C sensitivity in non-transformed cell strains derived from members of a cancer-prone family. *Carcinogenesis*, **12**, 1175-1180.

MIRZAYANS R, AUBIN RA, BLATTNER WA AND PATERSON MC. (1995). Abnormal pattern of post-gamma ray DNA replication in radioresistant fibroblast cell strains from affected members of a cancer-prone family with Li-Fraumeni syndrome. *Br. J. Cancer*, **71**, 1221-1230.

NEBERT DW. (1994). Drug-metabolizing enzymes in ligand-modulated transcription. *Biochem. Pharmacol.*, **47**, 25-37.

NOONAN KE, BECK C, HOLZMAYER TA, CHIN JE, WUNDER JS, ANDRULIS IL, GAZDAR AF, WILLMAN CL, GRIFFITH B, VON HOFF DD AND RONINSON IB. (1990). Quantitative analysis of MDR1 (multidrug resistance) gene expression in human tumors by polymerase chain reaction. *Proc. Natl Acad. Sci. USA*, **87**, 7160-7164.

PATERSON MC, MIDDLESTADT MV, WEINFELD M, MIRZAYANS R AND GENTNER NR. (1986). Human cancer-prone disorders, abnormal carcinogen response, and defective DNA metabolism. In: *Radiation Carcinogenesis and DNA Alterations*, Burns FJ, Upton AC and Silini G (eds) pp. 471-498. Plenum Press: New York.

RILEY RJ AND WORKMAN P. (1992). DT-diaphorase and cancer chemotherapy. *Biochem. Pharmacol.*, **43**, 1657-1669.

ROSS D, SIEGEL S, BEALL H, PRAKASH AS, MULCAHY RT AND GIBSON NW. (1993). DT-diaphorase in activation and detoxification of quinones. *Cancer Metastasis Rev.*, **12**, 83-101.

ROSVOLD EA, MCGLYNN KA, LUSTBADER ED AND BUETOW KH. (1993). Identification of an NAD(P)H:quinone oxidoreductase polymorphism and its association with lung cancer. *Proc. Am. Assoc. Cancer Res.*, **34**, 144.

SAMBROOK J, FRITSCH EF AND MANIATIS M. (1989). *Molecular Cloning: A Laboratory Manual*. Cold Spring Harbor Laboratory Press: Cold Spring Harbor, NY.

SARTORELLI AC. (1988). Therapeutic attack of hypoxic cells of solid tumours: presidential address. *Cancer Res.*, **48**, 775-778.

SIEGEL D, BEALL H, SENEKOWITSCH C, KASAI M, ARAI H, GIBSON NW, AND ROSS D. (1992). Bioreductive activation of mitomycin C by DT-diaphorase. *Biochemistry*, **31**, 7879-7885.

STROBEL HW AND DIGNAM JD. (1978). Purification and properties of NAD(P)H-cytochrome P-450 reductase. *Methods Enzymol.*, **52**, 89-96.

TALALAY P AND BENSON AM. (1982). Elevation of quinone reductase activity by anticarcinogenic antioxidants. *Adv. Enz. Regul.*, **20**, 287-300.

THOR H, SMITH MT, HARZELL P, BELLOMO G, JEWELL SA AND ORRENIUS S. (1982). The metabolism of menadione (2-methyl-1,4-naphthoquinone) by isolated hepatocytes. A study of the implications of oxidative stress in intact cells. *J. Biol. Chem.*, **257**, 12419-12425.

TOMASZ M, CHAWLA AK AND LIPMAN R. (1988*a*). Mechanism of monofunctional and bifunctional alkylation of DNA by mitomycin C. *Biochemistry*, **27**, 3182-3187.

TOMASZ M, LIPMAN R, MCGUINNESS BF AND NAKANISHI K. (1988*b*). Isolation and characterization of a major adduct between mitomycin C and DNA. *J. Am. Chem. Soc.*, **110**, 5892-5896.

TRAVER RD, HORIKOSHI T, DANENBERG KD, STADLBAUER THW, DANENBERG PV, ROSS D AND GIBSON NW. (1992). NAD(P)H:quinone oxidoreductase gene expression in human colon carcinoma cells: characterization of a mutation which modulates DT-diaphorase activity and mitomycin sensitivity. *Cancer Res.*, **52**, 797-802.

WINSHIP PR. (1989). An improved method for directly sequencing PCR amplified material using dimethyl sulphoxide. *Nucleic Acids Res.*, **17**, 1266.